# Rehabilitation Through Physical Activity and Sport in Light of the International Classification of Functioning, Disability, and Health–Current Research Perspectives

**DOI:** 10.3389/fresc.2022.840850

**Published:** 2022-04-28

**Authors:** Isabel Stolz, Elisa Weber, Ruud Vreuls, Volker Anneken

**Affiliations:** ^1^Research Institute for Inclusion Through Physical Activity and Sport, Affiliated Institute of the German Sport University Cologne, Frechen, Germany; ^2^Institute for Movement-and Neurosciences, German Sport University Cologne, Cologne, Germany

**Keywords:** exercise, functioning, ICF, physical activity, rehabilitation, sports

## Abstract

The implementation of functioning by the World Health Organization (WHO) as the third global health indicator, along with mortality and morbidity, represents a promising advancement for a comprehensive assessment of international health systems and health strategies. The description of a person's health state operationalized by both biological and lived health *via* functioning provides a holistic picture of an individual's life situation and proved to be successful in building a framework for formulating therapy goals, achievable activities, and participational aspects against the background of an individual's life situation. Furthermore, improving an individual's functional ability and wellbeing could potentially affect the health indicators of morbidity and mortality and will be codable beyond the ICF in ICD-11. This methodological perspective emphasizes the use of ICF applications on the wider and narrower level of international rehabilitation systems and highlights the incorporation of the term functioning in rehabilitation through physical activity and sport. Current research perspectives in applying the ICF and functioning in clinical and rehabilitation practices are discussed and a current explorative study is presented, which applies the holistic orientation of functioning and the biopsychosocial model to the specific case by an individualized sports coaching intervention in rehabilitation. Subsequently, a unifying ICF- oriented language in rehabilitation is considered as a powerful foundation for a consistent international research strategy concerning increased international collaborations and future research perspectives.

## Introduction

In May 2001, the World health assembly adopted the International Classification of Functioning, Disability and Health (ICF) (1), which is intended to enable internationally consistent terminology about the impacts of health problems, taking into account a person's entire background (2). One goal of the ICF is the uniform understanding of the impact of health problems on an individual's health status. Therefore, the ICF's biopsychosocial model defines a person's health state through the person's biological health combined with the person's “lived health” interacting with personal and environmental factors (3). The International Classification of Diseases (ICD) represents the international standard diagnostic classification for diseases and related health problems (4). It complements the ICF, to monitor the response of health systems to people's health needs from a bio-medical perspective with the two indicators of mortality and morbidity (5). Mortality describes the length of life of a population and its survival with health problems. Morbidity, on the other hand, describes the distribution of health conditions in the population (5). However, in order to fully describe people's health needs, a third indicator was necessary that links biological and lived health: functioning. This indicator has also been coded in ICD-11, published in January 2022, and is linked to the ICF (5). On the one hand, functioning can describe the health status of a population and the outcome of clinical interventions; on the other hand, it can depict and monitor the impact of health system services on the lived health experience of individuals (6). The result shows an increased permeability in international health care systems using ICD-11. Furthermore, this outcome enables that ICF classifications will be feasible in the future. The purpose of this article is 2-fold. First, current research perspectives are discussed in applying the ICF on human functioning in clinical and rehabilitation practices. Second, an ICF-based research approach is presented by means of physical activity and sport. The article aims to contribute to the discussion of where we currently stand in terms of functioning-orientated rehabilitation and to provide perspectives on how to improve a person's lived experience of health based on the holistic view of the ICF framework.

## Rehabilitation in Light of the ICF

Considering aging population and the increasing prevalence of chronic health problems, world health systems face new demographic challenges. For this reason, there is a global need to invest in improving individuals' functional ability and wellbeing in addition to reducing morbidity and mortality (7, 8). In this regard, functioning is the key indicator for rehabilitation and aims to optimize the functioning of persons who have or are likely to experience an impairment in function and to change the state of disability in order to achieve activities and participation (7, 9–12). In order to compare outcomes, efficiency and cost-effectiveness of different rehabilitation interventions, differences between the change in functioning after completion of a particular intervention compared to another intervention must be discussed (13). To compare rehabilitation interventions and provide standardized reporting, the ICF stands at the forefront of the rehabilitation community (13). According to Stucki et al. (14) the ICF is a powerful health information reference system for documenting functioning in rehabilitation. Due to this importance and to counteract the demographic trend, there is a need for investment in rehabilitation systems. In order to achieve targeted rehabilitation goals, the dimensions of health, assistive devices, and accessibility must be aligned, so that people with disability can achieve participation in society and improve their quality of life. For example, by allied rehabilitation disciplines, that could enable person centered services, such as promoting health behavior in concert with mobility training and fitted assistive technology (e.g., hand bikes, wheelchairs, eHealth tools) (15).

Furthermore, it is necessary to improve existing assessment tools and to develop new ones to disseminate knowledge and to precisely depict a holistic view on a person's “lived health” in addition to their biological health for a targeted engagement of multi-professional teams in clinical and outpatient fields (10).

ICF applications have been implemented in the wider and narrower level of international rehabilitation systems in the past two decades. In this context, the ICF, with its holistic orientation, was partly used as an underlying theoretical framework, partly operationalized at the specific item level as an assessment or used to define therapy goals with patients (16). In this regard, in the US-American area, the ICF was primarily used in its conceptual approach, whereas in the European region, the concrete application in clinical practice was focused (ibid.). The ICF has been successfully integrated, for instance, into electronic health records (EHRs) in past years. Results of a review by Maritz et al. (17) showed increased comprehensiveness and interdisciplinary focus when the ICF was used in EHRs (17). Also, numerous ICF-core sets have been developed and published on the ICF Research Branch platform to provide a basis for a standardized international assessment focusing on human functioning in clinical contexts (18). Furthermore, a family-friendly ICF version for common language in communicating with parents and professionals has been developed and validated in four European countries, to ease ICF language barriers for multidisciplinary collaboration and cooperation with parents and care-givers (19). These developments provide a strong foundation for the scientific community and represent a promising future for a more functional view of patient's heath states. In this regard the central question of the 2021 Rehabilitation International Congress was therefore: Where do we currently stand and which research strategies would apply to rehabilitation practice and heath care to make optimal improvements of a person's “lived health” by focusing on functioning. Concerning this, current ICF-based applications and potential future research directions were discussed on the conference and are presented in the following paragraph.

## Results of the 2021 Rehabilitation International Congress

At the Rehabilitation International Congress 2021 in Aarhus numerous ICF-based applications were presented and indicators for potential future research directions were given, that in particularly highlighted the digital processing and use of the ICF-classification. Decker (20) presented the REHADAT-ICF platform as a practical research tool to find and share targeted information about rehabilitation, vocational inclusion, and assistive technology in the cross-national ICF structure (20). Bjørnshave et al. (21) discussed the ICF model as a communication tool with patients. Results have shown that the ICF component interactions proved promising when talking with patients about complex issues in their sphere of life. Ng et al. (22) presented an ICF application in stroke rehabilitation which enhanced communication in a multi-disciplinary team and showed promise in guiding the design of personal and holistic rehabilitation plans (22). Chan (23) also stressed the benefits of the ICF's systematic and individual person-centered service framework, which facilitated trans-disciplinary collaboration and synergy in joint professional case conferences (23). Additional innovative rehabilitation concepts were presented, such as outdoor rehabilitation programs, with no direct link to the ICF classification. Results by Alfredssen (24) indicated that an outdoor rehabilitation program empowered patients to go outside on their own. It therefore could indirectly prepare the post-rehabilitation phase and promote participation in daily life routines for patients. Autrup and Glümer (25) found that individuals had more energy after outdoor classes, and inactive, isolated, and vulnerable individuals became an integrated part of the group through these interventions. Also, results indicated that outdoor-only rehabilitation programs increased self-management and empowerment (25). In this regard, it would also be of interest to examine, on an ICF basis, to what extent these demanding environmental conditions affect activities, participation, health state, and which role physical activity and sport played in the processes.

Mentioned studies confirm the global trend that there is currently a growing number of high-quality ICF-related developments in rehabilitation contexts (26–28). However, the role of physical activity and sport represents an important variable that seems to play a subordinate role in the current discourse. As the contributions to outdoor rehabilitation have shown, impact factors of sport and exercise can make a major contribution to enhance persons' biomedical functioning, feasible activities, and social participation opportunities (29–31). In this context, aspects of training and the increase in and maintenance of physical performance play a role in the psychosocial promotion of getting active independently, self-determination, and experiencing self-efficacy (32). These are particularly important in terms of rehabilitation goals in the context of returning to work, as they enable people to regain access to working life (33).

## Pilot Study “Sportcoach”

In this context, a project of the German social accident insurance institution for the health and welfare services (BGW), as evaluated by the Research Institute for Inclusion through Physical Activity and Sport, is presented. The Sportcoach-study aims to improve the quality of life, self-efficacy, fitness, and participation of persons with work-related polytrauma and occupational diseases through an individualized sports coaching approach in rehabilitation. The project runs as a pilot study in six regions in Germany. The aim is to achieve and sustain participation in social and working life of persons with occupational diseases through physical training with sports coaches and the involvement in sporting activities and participation in regional sport clubs. The sports coach advises, accompanies, and mediates persons into sporting activities and participation in sport clubs. The participants benefit from these coaching services for 18 months, in which they can try out various sporting activities and enroll in sport clubs. The selection of these sporting activities depends on numerous external and internal factors, such as distance from home and personal sport preferences. Furthermore, the coach is allocated to a person, after they have completed their medical rehabilitation at their local hospital. In the ICF biopsychosocial structure, the coach can be understood as a promoting environmental factor who guides persons with polytrauma and occupational diseases toward a healthier lifestyle post-rehabilitation to increase their global functioning capacities. The coach aims to follow the main goal of rehabilitation by a holistic approach and to optimize functioning for an enhanced health state *via* an individualized approach.

The coach's first task is to find appropriate sporting activities close to a patient's regional environment and residence; the sports are highly suited for this particular person and their individual biomedical background. As a result, the coach's approach is somewhat different from the established and widely known rehabilitation interventions. Most rehabilitation interventions focus on the increase of one particular health topic, such as the relief of dorsal pain. Furthermore, these interventions, also known as rehabilitation sport classes, are created in such a way that a wide spectrum of persons can attend these courses. This means that these classes possess a low threshold to participation and the subject matter is shortened to limited practices. However, because of this general approach, this kind of practice is effective for only a certain group of individuals and does not promote long-term involvement in sport groups close to their home. Therefore, the coach tries to find sporting activities which are highly suited to the participants' personal health needs and which promote a long-term involvement in the sport structures in their regional environment. Aside from that, the other main criterion for the coach's approach is to support individuals to participate in sport programs at sport clubs. By doing so, the effect can be 2-fold. First, participants are able to increase their health state further, since these sport activities have a higher personal binding and are guided by professionals (body functions and –structures, activities). Second, by attending sport activities at sport clubs, participants can exercise with people who share the same passion about that particular activity and have similar functioning levels in sport and thus be involved with others (participation). This effect can lead to various actions on the social level, such as building new friendships or to become a permanent member at the club. The overarching goal can be the increased participation through sports, because being involved in this sporting area may open up other potential pathways for participation in society more easily.

Since the study is still ongoing, most participants, *n* = 71, only completed two measurement points. However, preliminary results after 5 months, from t1 to t2, of the coach's approach show qualitative and quantitative effects in several areas. First, all participants reported that they were satisfied with the coaches' individualized approaches. Most notably, the participants described that they appreciated their motivational manner and their ability to recognize their personal needs. Furthermore, first quantitative results show an increase in subjective activity and health levels using the perceived physical condition scale (peps) (34). Additionally, similar effects were found, analyzing the quality of life scale (QOLS) (35). The results highlight a potential increase on the physical and social subscales. These preliminary results demonstrate that the coach's approach could be useful as a new ICF-based concept to further optimize holistic rehabilitation's aim by using a sport-orientated functioning approach. However, the above-mentioned results should be interpreted with caution, since the data only reflects the first impressions of the coach's approach. Therefore, further data is needed in order to confirm these first findings on the basis of larger sample sizes.

## Discussion

In summary, the sports coach approach represents an example of the concrete application of the ICF framework, which applies the holistic orientation of functioning and the biopsychosocial model to the individual case. Against the backdrop of the global situation described at the beginning of this article, individualization will become an increasingly important rehabilitation issue in the future. The establishment and anchoring of functioning in the ICF and ICD-11 was a required step in order to provide a complete set of indicators to monitor, compare, and align all five WHO health strategies (preventive, promotive, curative, rehabilitative, and palliative). In addition, it is important to compare and contrast data in international research collaborations, in which physical activity and sport play a significant role (12). The revision of ICD-11 has also greatly simplified the usage and improved the user-friendliness of the classification. There are now also online platforms available, where international contribution has been made possible (36). In addition, consistency between language versions has been improved and a Unique Reference Identifier (URI) has been developed, which for the first time represents a unique string of characters for individualized cases. This URI enables connectivity and linkage to holistic rehabilitation concepts (ibid.). The ICF has brought a new perspective to the health condition of a person, which, on the basis of the findings presented, can be depicted more and more precisely and better linked (37). Through the increasing implementation of digital health records, the ICF-holistic view of a person's functioning can build a basis to realize the optimal treatment for a certain health condition in multi-professional teams. As Wenzel and Morfeld (16) point out for rehabilitation teams working with ICF checklists, the participative orientation of their work as well as interdisciplinary collaboration and a generally more systematic way of approaching work improved. Furthermore, ICF applications proved themselves to be suitable in examination schedule, the identification of relevant functional areas and therapy target formulations (ibid.). Prospectively, there will also be an increase in assistive technologies and devices in rehabilitation; for example, for barrier-free movement, individualized physical training strategies or complementary eHealth Coaching. In this regard, the professional field in rehabilitation is becoming more diverse (including bio technicians, engineers, sports scientists) and it is becoming increasingly important that functioning goals and outcomes are defined precisely and comprehensively. Statistical mapping and structural equitation modeling could also provide further insights in the complexity of ICF-interactions in regard to human functioning (37). In this regard, a unifying ICF-oriented language in rehabilitation provides a powerful foundation for consistent international research strategies and common efforts of the creation of new knowledge (ibid.). The use of functioning as an indicator for health can be a guiding principle in this respect, as it focuses on enhancement of the person's lived health, which could be realized by tailored and functionally supportive environmental factors such as tech devices and assistive technologies in a targeted way.

## Data Availability Statement

The original contributions presented in the study are included in the article/supplementary files, further inquiries can be directed to the corresponding authors.

## Ethics Statement

The studies involving human participants were reviewed and approved by Ethics Committee German Sport University Cologne. The patients/participants provided their written informed consent to participate in this study.

## Author Contributions

IS and EW devised the article structure and the conceptual framework. RV contributed to the conceptual idea and discussed study findings in this work. VA supervised the manuscript process. All authors discussed the results and contributed to the final manuscript.

## Conflict of Interest

The authors declare that the research was conducted in the absence of any commercial or financial relationships that could be construed as a potential conflict of interest.

## Publisher's Note

All claims expressed in this article are solely those of the authors and do not necessarily represent those of their affiliated organizations, or those of the publisher, the editors and the reviewers. Any product that may be evaluated in this article, or claim that may be made by its manufacturer, is not guaranteed or endorsed by the publisher.
